# Optical Stability of Injectable Resin Composites with Different Shade-Matching Strategies

**DOI:** 10.3390/polym18212736

**Published:** 2026-11-03

**Authors:** Buse Kesgin, Başak Yazkan

**Affiliations:** Department of Restorative Dentistry, Faculty of Dentistry, Mugla Sıtkı Koçman University, Mugla 48000, Türkiye; busekesgin@gmail.com

**Keywords:** injectable resin composite, shade-matching strategy, color stability, whiteness index, translucency, bleaching

## Abstract

This study evaluated the effects of aging, coffee staining, and bleaching on the optical properties of injectable resin composites with varying filler types, resin matrix compositions, and shade-matching strategies. Six injectable resin composites and one conventional microhybrid composite were evaluated (*n* = 20 per material; 140 specimens in total). For each material, specimens were randomly allocated to two subgroups (*n* = 10): thermocycling or 12-day coffee staining followed by in-office bleaching. Disc-shaped specimens were fabricated, and baseline color, whiteness index, and translucency were recorded. Data were analyzed using repeated-measures ANOVA and one-way ANOVA, followed by Tukey’s HSD post hoc test for between-material comparisons (α = 0.05). Significant differences among materials were observed after aging, staining, and bleaching (*p* < 0.001). After aging, the multi-shade nanofilled FSF, group-shade nanohybrid NSF, and conventional microhybrid GDA showed the lowest color changes, whereas the one-shade composites showed the highest. Coffee staining reduced whiteness and translucency in all groups, with the greatest whiteness loss observed in the one-shade composites. Bleaching reduced staining-induced discoloration in all materials, most markedly in the one-shade nanohybrid CBF; however, residual color differences relative to baseline remained above the acceptability threshold in all materials. After bleaching, translucency in the supra-nanospherical OBF and EBF composites exceeded baseline values beyond the perceptibility threshold. Within the limitations of this in vitro study, the evaluated resin composites exhibited material-dependent changes in color, whiteness, and translucency following artificial aging, coffee staining, and bleaching, with incomplete recovery of baseline optical properties after bleaching.

## 1. Introduction

Direct anterior restorations represent a cornerstone of contemporary restorative dentistry, driven by increasing esthetic demands and continuous advances in adhesive and restorative materials. Although the conventional incremental layering technique remains widely used, it is associated with several limitations, including extended chair time, the risk of air entrapment between layers, and compromised interlayer bonding resulting from procedural contamination [1]. In response to these limitations, the injectable composite technique has emerged as a minimally invasive and clinically efficient alternative, allowing for improved flowability, enhanced cavity adaptation, and simplified placement protocols [2,3]. Beyond handling advantages, injectable resin composites have increasingly been promoted for their esthetic potential in anterior restorations, where optical integration and shade adaptation are critical. Nevertheless, the long-term clinical success of injectable restorations remains highly dependent on the optical and physicochemical properties of the composite materials employed [4].

Contemporary injectable resin composites employ different shade-matching strategies, including conventional multi-shade pigment-based systems and simplified group-shade or one-shade concepts that rely on optical blending and structural color mechanisms to harmonize with surrounding tooth structure. Color stability and accurate shade matching are fundamental determinants of the long-term esthetic performance of anterior composite restorations [3]. Injectable composites designed to simplify shade selection are expected to visually integrate with surrounding enamel and dentin through controlled translucency and light diffusion rather than precise pigment matching. Consequently, their optical behavior may differ substantially from that of conventional pigment-based composites when exposed to clinical challenges over time [1,4].

Both intrinsic and extrinsic factors such as degree of polymerization, water sorption, surface roughness, finishing and polishing procedures, and dietary habits have been shown to significantly influence the color stability of resin composites [5]. In addition, material-related parameters, including filler type, filler size, filler loading, and degree of conversion, play a decisive role in determining optical and mechanical performance [6]. Injectable resin composites often exhibit modified filler types or reduced filler content to enhance flowability, which may compromise resin–filler interfacial integrity. Insufficient interfacial bonding can increase water sorption and facilitate stain penetration, ultimately leading to discoloration [7]. These effects may be particularly relevant for injectable composites employing simplified shade-matching strategies, where subtle alterations in light transmission may disproportionately affect perceived color adaptation.

Translucency represents a key optical property required to replicate the depth and vitality of natural tooth structure. Light interaction within resin composites is governed by reflection, transmission, absorption, and scattering at the filler–matrix interface. Variations in filler morphology, refractive index mismatch, and filler distribution influence light diffusion within the material and determine translucency and overall esthetic integration [8]. Unlike conventional pigment-based composites, which achieve shade matching primarily through selective light absorption, one-shade and group-shade injectable composites depend largely on light scattering, translucency modulation, and structural color effects. As a result, their optical behavior may be more sensitive to aging, staining, and bleaching challenges. In recent years, continuous modifications in resin matrix formulations and filler technologies have aimed to enhance both optical and mechanical properties, particularly in composites designed for esthetic anterior applications [8,9]. However, the extent to which these innovations improve the long-term optical stability of injectable composites with different shade-matching concepts remains unclear [9].

Bleaching procedures may affect the optical properties of resin composites by inducing surface degradation, altering the resin–filler interface, and modifying light transmission characteristics [10,11]. Although the effects of bleaching agents on conventional resin composites have been extensively investigated, evidence regarding injectable resin composites, especially those incorporating novel filler technologies and simplified shade-matching strategies, remains limited [12,13,14,15,16]. Given the increasing use of injectable composites in highly esthetic regions, understanding their response to bleaching after staining is of particular clinical relevance.

Despite growing clinical adoption, data on the behavior of injectable resin composites after aging and after staining followed by bleaching remain scarce. While clinical case reports have described discoloration and esthetic degradation following prolonged intraoral exposure, systematic investigations evaluating how aging and bleaching procedures influence the optical properties of injectable composites are lacking [17,18,19]. Moreover, the role of filler type and shade-matching strategy in color stability, translucency, and whiteness following these challenges has not been adequately clarified. Given the increasing clinical reliance on injectable resin composites for highly esthetic anterior restorations, clarifying whether simplified shade-matching concepts preserve optical stability to a degree comparable with conventional pigment-based systems is essential for material selection and long-term esthetic predictability.

Therefore, the aim of the present study was to evaluate the effects of aging, staining, and bleaching on the optical properties of injectable resin composites with different filler types and shade-matching strategies.

The null hypotheses tested were as follows:

(1) Coffee staining does not result in statistically significant differences in color change (ΔE_00_), translucency change (ΔTP), or whiteness difference (ΔWID) among injectable resin composites employing different filler types and shade-matching strategies.
(2) Artificial aging does not cause statistically significant changes in ΔE_00_, ΔTP, or ΔWID among injectable resin composites employing different filler types and shade-matching strategies.
(3) Bleaching following coffee staining does not significantly alter ΔE_00_, ΔTP, or ΔWID values of injectable resin composites employing different filler types and shade-matching strategies.

## 2. Materials and Methods

### 2.1. Materials

Six injectable resin composites with different shade-matching capabilities and filler types and one conventional microhybrid composite were evaluated in the present study. The shade-matching strategy, shade, group code, filler type, composition, filler size, filler content (wt%/vol%), manufacturer, and lot number of each material are listed in Table 1.

**Figure 1:**
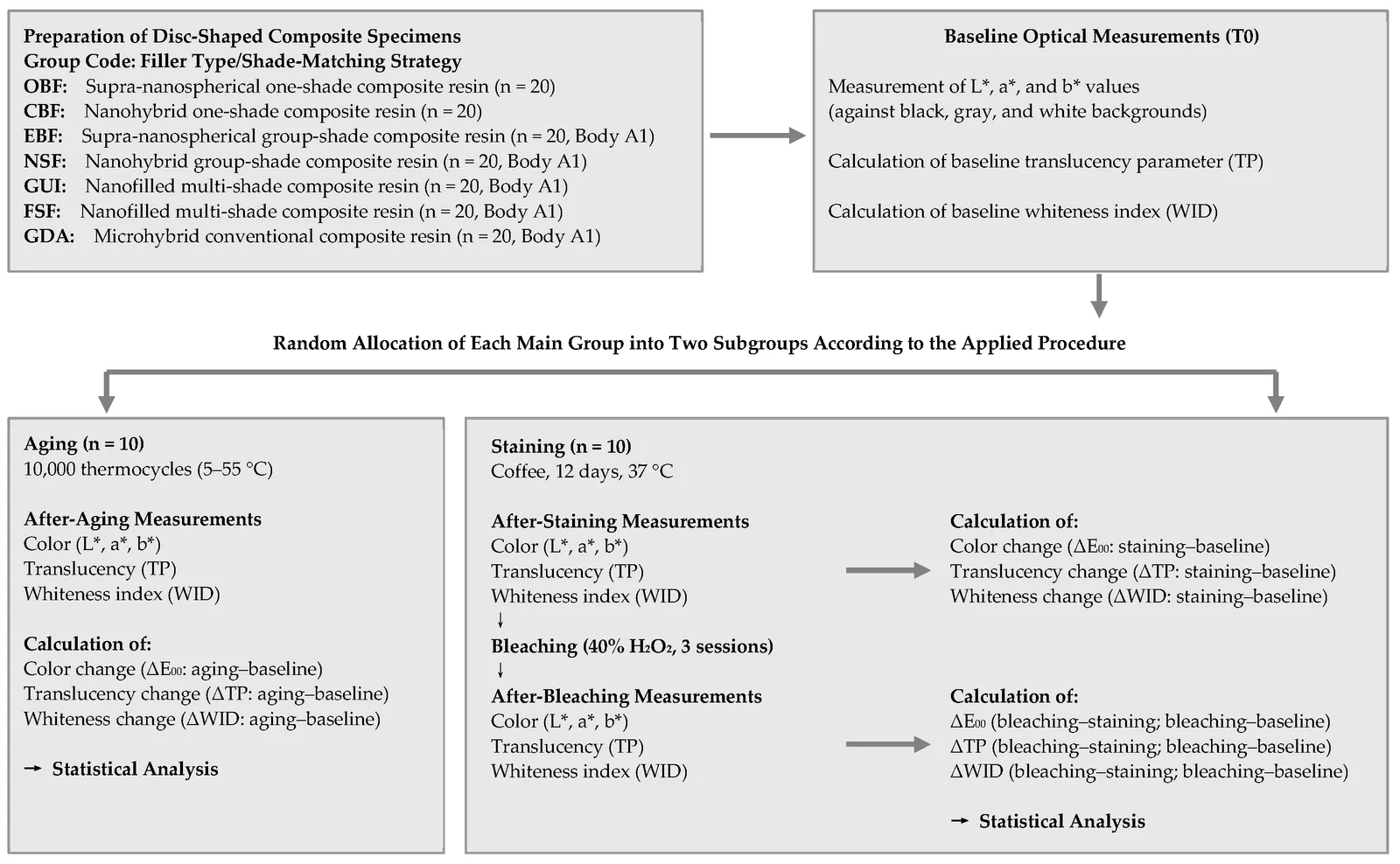
Flowchart of the study protocol. OBF, Omnichroma Bulk Fill Flow; CBF, Charisma Bulk Flow ONE; EBF, Estelite Bulk Fill Flow; NSF, Neo Spectra ST Flow; GUI, G-ænial Universal Injectable; FSF, Filtek Supreme Flowable Restorative; GDA, Gradia Direct Anterior; TP, translucency parameter; WID, whiteness index for dentistry; T0, baseline.

A flowchart summarizing the study protocol is shown in Figure 1.

### 2.2. Specimen Preparation

A total of 20 specimens were fabricated for each group using cylindrical Teflon molds (8 mm in diameter and 2 mm in thickness). The restorative materials were placed between Mylar strips and glass slides to produce standardized, flat surfaces. Each specimen was light-cured for 20 s using an LED curing unit (VALO Grand, Ultradent, South Jordan, UT, USA) with an irradiance of 1200 mW/cm^2^, in accordance with the manufacturers’ instructions. After curing, specimens were stored in distilled water at 37 °C for 24 h to allow post-polymerization maturation. This 24 h storage period was implemented to obtain a mechanically and optically stable surface, ensuring that subsequent polishing and optical measurements reflected material-related properties rather than early polymerization artifacts. The upper surfaces of all resin composite discs were then subjected to a standardized finishing and polishing protocol. Prior to finishing and polishing, the thickness of each specimen was measured at three different points using a dial caliper (Mitutoyo 505-667, Mitutoyo Corporation, Kawasaki, Japan; resolution 0.01 mm), and only specimens with a thickness of 2.0 ± 0.05 mm were included; specimens outside this range were replaced. Finishing and polishing procedures were performed to obtain a uniform and clinically relevant surface condition prior to baseline optical measurements, as follows:

Initial finishing was carried out using aluminum oxide abrasive discs (Sof-Lex™ Pop-On, 3M ESPE, St. Paul, MN, USA) in a sequential manner (medium, fine, and extra-fine grits). Each disc was applied for 15 s under constant water cooling using a low-speed handpiece operating at approximately 10,000 rpm. Light, intermittent pressure was applied in a unidirectional motion, and the direction of polishing was alternated between successive steps to ensure homogeneous surface treatment.

Subsequently, final polishing was performed using a multi-step diamond-impregnated polishing system designed for anterior resin composites (Enhance™ followed by PoGo™, Dentsply Sirona, Konstanz, Germany). Polishing was conducted under dry conditions at 8000 rpm with minimal pressure for 20 s per step to achieve a high-gloss surface and minimize heat generation. Between each polishing step, specimens were thoroughly rinsed with distilled water and gently air-dried.

All finishing and polishing procedures were performed by a single experienced operator (BK) to eliminate inter-operator variability. Following polishing, specimens were ultrasonically cleaned in distilled water for 5 min to remove residual abrasive particles and surface contaminants. The polished surfaces were visually inspected under standardized lighting conditions to confirm surface uniformity before further analyses.

### 2.3. Baseline Optical Evaluation

The L*, a*, and b* values were measured at baseline (T0) under standardized D65 illumination using a calibrated dental spectrophotometer (VITA Easyshade V; VITA Zahnfabrik, Bad Säckingen, Germany) according to the CIE L*a*b* color system and recorded. Color measurements were performed against a standardized gray background for the evaluation of color stability and whiteness index changes, and against black and white backgrounds for translucency assessment. The device was calibrated before each measurement session in accordance with the manufacturer’s instructions. During measurements, the spectrophotometer probe was positioned perpendicular to the center of each specimen to minimize edge effects and ensure consistent light capture. For each specimen, three consecutive measurements were obtained, and the mean values were calculated and used for subsequent analyses.

Baseline whiteness index for dentistry (WID) values were calculated using the following equation [20]:

(1) *WID* = 0.511*L* ∗ − 2.324*a* ∗ − 1.100*b* ∗

The baseline translucency parameters (TP) were calculated as the color difference between measurements obtained over white (w) and black (b) backgrounds according to the following equation [21]:

(2) $$\mathrm{TP}\;=\;\sqrt{{\left({L*}_{w}\;-\;{L*}_{b}\right)}^{2}\;+\;{\left({a*}_{w}\;-\;{a*}_{b}\right)}^{2}\;+\;{\left({b*}_{w}\;-\;{b*}_{b}\right)}^{2}}$$

### 2.4. Randomization of Composite Discs

For each resin composite, 20 specimens were allocated to two experimental subgroups (aging and staining/bleaching) using a restricted randomization procedure. A mobile dice-rolling application configured to generate two possible outcomes (1 or 2) was used for specimen allocation. A separate virtual dice roll was performed for each specimen, and allocation was determined according to the generated outcome. Once one subgroup reached 10 specimens, the remaining specimens were assigned to the other subgroup to ensure equal sample sizes (*n* = 10 per subgroup). The specimens were allocated to two independent experimental subgroups to separately assess the optical effects of thermal aging and coffee staining followed by bleaching. This allocation enabled the evaluation of each experimental challenge without the potential confounding influence of prior thermal aging on staining and bleaching outcomes.

### 2.5. Group A: Aging

Thermal aging was performed using a thermocycling protocol to simulate intraoral thermal stresses associated with daily consumption of hot and cold beverages. Specimens were subjected to 10,000 thermocycles between water baths maintained at 5 °C and 55 °C, representing approximately one year of clinical service [22]. Each cycle consisted of a dwell time of 15 s in each bath, with a transfer time of 5 s between baths.

Upon completion of the aging protocol, specimens were removed from the thermocycling device, gently dried, and allowed to equilibrate at room temperature for 1 h prior to optical measurements. Color coordinates, whiteness index, and translucency parameter values were then remeasured and recorded as T1.

### 2.6. Group B: Staining and Bleaching

#### 2.6.1. Staining

Specimens were immersed in a coffee solution prepared by dissolving 2 g of coffee powder in 200 mL of boiled distilled water and allowing it to cool to room temperature before use. The specimens were stored in the staining solution for 12 consecutive days at 37 °C to simulate approximately one year of clinical staining exposure. To ensure consistent staining conditions, the coffee solution was freshly prepared and renewed daily. During the staining period, specimens were kept fully immersed without agitation [23].

At the end of the staining protocol, specimens were rinsed thoroughly with distilled water for 30 s to remove superficial residues, gently air-dried, and allowed to equilibrate at room temperature for 1 h prior to optical measurements. Optical measurements were then performed and recorded as T1.

#### 2.6.2. Bleaching

Following staining, an in-office bleaching protocol was applied using a 40% hydrogen peroxide gel (Opalescence Boost; Ultradent, South Jordan, UT, USA). The bleaching agent was applied uniformly to the specimen surfaces in an approximately 1-mm-thick layer. Manual activation was performed at 5-min intervals during two consecutive 20-min applications per session, in accordance with the manufacturer’s recommendations. The bleaching procedure was conducted in three separate sessions on days 1, 8, and 15 to simulate a commonly used clinical in-office bleaching regimen [24].

After each bleaching session, specimens were rinsed thoroughly with distilled water to remove residual bleaching gel. Upon completion of the final session, specimens were gently air-dried and allowed to equilibrate at room temperature for 1 h prior to optical measurements. Optical measurements were repeated and recorded as T2.

### 2.7. Optical Outcome Parameters and Thresholds

Color change (ΔE_00_) was calculated using the CIEDE2000 formula [25,26], which provides improved correlation with visual perception by accounting for nonuniformity in the CIE L*a*b* color space, according to the following equation:

(3) $${\Delta E}_{00}=\sqrt{{\left(\frac{\Delta L^{\prime }}{k_{L}S_{L}}\right)}^{2}+{\left(\frac{\Delta C^{\prime }}{k_{C}S_{C}}\right)}^{2}+{\left(\frac{\Delta H^{\prime }}{k_{H}S_{H}}\right)}^{2}+R_{T}\left(\frac{\Delta C^{\prime }}{k_{C}S_{C}}\right)\left(\frac{\Delta H^{\prime }}{k_{H}S_{H}}\right)}$$

where ΔL′, ΔC′, and ΔH′ are the differences in lightness, chroma, and hue between the two measurements; S_L_, S_C_, and S_H_ are the weighting functions for the lightness, chroma, and hue components, respectively; R_T_ is the rotation function that accounts for the interaction between chroma and hue differences in the blue region; and k_L_, k_C_, and k_H_ are the parametric factors, which were set to 1 in the present study [25,26].

Color differences were calculated for three intervals: between baseline and post-staining measurements (ΔE_00_ Staining–Baseline), between post-staining and post-bleaching measurements (ΔE_00_ Bleaching–Staining), and between baseline and post-bleaching measurements (ΔE_00_ Bleaching–Baseline). ΔE_00_ Staining–Baseline represents the magnitude of discoloration induced by staining, ΔE_00_ Bleaching–Staining represents the magnitude of color change produced by bleaching, and ΔE_00_ Bleaching–Baseline represents the residual color difference relative to baseline. Because ΔE_00_ is a non-directional measure, the direction of color changes was characterized by the mean differences in CIELAB coordinates (ΔL*, Δa*, Δb*) and by ΔWID. A reduction in discoloration after bleaching was defined as a lower ΔE_00_ Bleaching–Baseline value compared with the ΔE_00_ Staining–Baseline value, whereas the color was considered to have returned to an acceptable level only when ΔE_00_ Bleaching–Baseline was below the acceptability threshold. Similarly, ΔTP Bleaching–Baseline values within ±2 units were considered not perceptibly different from baseline.

Color differences were interpreted using established perceptibility and acceptability thresholds of ΔE_00_ = 0.8 and 1.8, respectively, corresponding to the minimum perceptible and maximum clinically acceptable color differences for 50% of observers under controlled conditions [27].

Whiteness changes were expressed as whiteness difference (ΔWID), calculated as the difference between post-treatment and baseline WID values (ΔWID = WID_after_ − WID_baseline_). Interpretation of ΔWID values was based on previously reported perceptibility and acceptability thresholds of 0.72 and 2.60 units, respectively, according to a 50:50 observer criterion [28].

Changes in translucency were expressed as differences in the translucency parameter (ΔTP), calculated by comparing values obtained at baseline (T0), after aging or staining (T1), and after bleaching (T2). A ΔTP value of 2 units was considered the visual perceptibility threshold, corresponding to a contrast ratio difference (ΔCR) of 0.07, with values exceeding this threshold regarded as visually perceptible under clinical viewing conditions [29].

### 2.8. Statistical Analysis

Statistical analyses were performed using SPSS Statistics, version 31 (IBM Corp., Armonk, NY, USA). The normality of data distribution was assessed using the Shapiro–Wilk test, and homogeneity of variances was evaluated using Levene’s test. Two separate levels of analysis were performed.

Within-material changes: For each material, changes in WID and TP values across treatment stages (baseline T0, after aging or staining T1, and after bleaching T2) were analyzed using repeated-measures ANOVA, followed by Bonferroni-adjusted pairwise comparisons. When the assumption of sphericity was violated, the Greenhouse–Geisser correction was applied. Since ΔE_00_ represents the color difference between two measurements, within-material color changes were interpreted with respect to the perceptibility and acceptability thresholds described in Section 2.7.

Between-material differences: Difference values (ΔE_00_, ΔWID, and ΔTP) were calculated at the individual specimen level and compared among materials for each treatment interval using one-way ANOVA, followed by Tukey’s honestly significant difference (HSD) post hoc test. Data are presented as mean ± standard deviation, and the level of statistical significance was set at *p* < 0.05.

## 3. Results

### 3.1. Effects of Aging on Optical Properties

**Figure 2:**
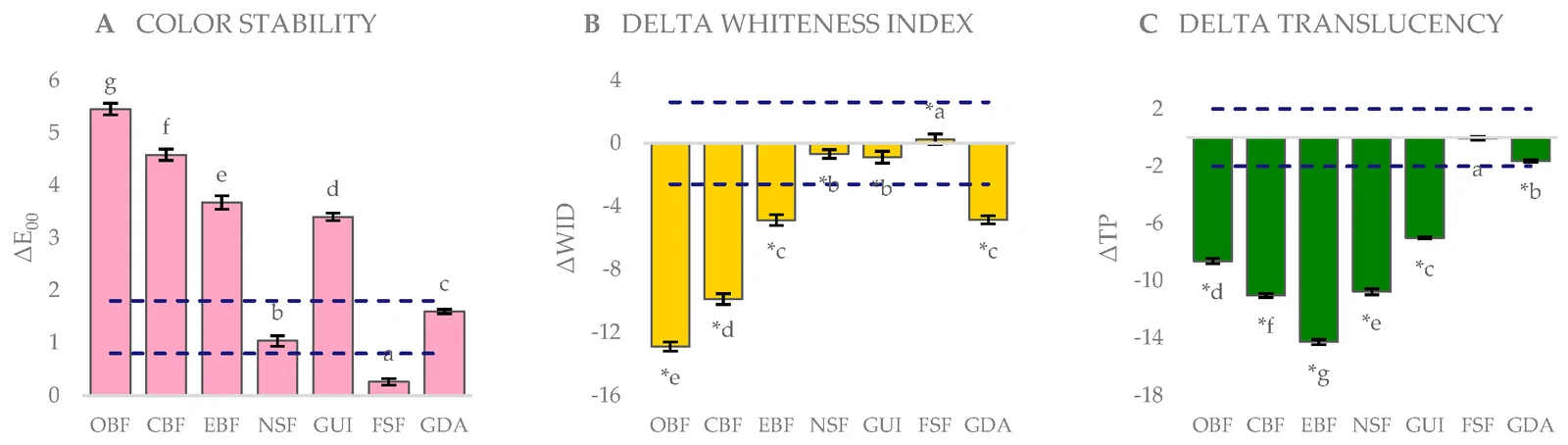
Optical changes of the tested resin composites after artificial aging (10,000 thermocycles) relative to baseline: (**A**) color change (ΔE_00_); (**B**) whiteness index difference (ΔWID); (**C**) translucency parameter change (ΔTP). Bars represent mean values and error bars standard deviations (*n* = 10). Dashed lines indicate the perceptibility and acceptability thresholds (ΔE_00_ = 0.8 and 1.8; ΔWID = ±2.60; ΔTP = ±2). Different lowercase letters indicate statistically significant differences among groups (one-way ANOVA followed by Tukey’s post hoc test, *p* < 0.05), with letters assigned in ascending order of the magnitude of change (a = smallest change); asterisks (*) in panels (**B**) and (**C**) indicate a statistically significant change from baseline.

Color change (ΔE_00_), whiteness difference (ΔWID), and translucency change (ΔTP) after aging relative to baseline are presented in Figure 2.

*Between-material comparisons*. After aging, significant differences were found among materials for ΔE_00_, ΔWID, and ΔTP (one-way ANOVA, *p* < 0.001; Figure 2). The highest ΔE_00_ was recorded for the one-shade supra-nanospherical OBF (5.45 ± 0.11), followed by the one-shade nanohybrid CBF (4.58 ± 0.11) and the group-shade supra-nanospherical EBF (3.67 ± 0.13), whereas the lowest ΔE_00_ was observed in the multi-shade nanofilled FSF (0.26 ± 0.06). The greatest reduction in whiteness was observed in OBF (−12.89 ± 0.29), followed by CBF (−9.88 ± 0.34), whereas FSF exhibited a slight increase (+0.26 ± 0.32). The largest decrease in translucency was found in EBF (−14.27 ± 0.18), followed by CBF (−11.01 ± 0.14) and the group-shade nanohybrid NSF (−10.76 ± 0.21), whereas the smallest change was observed in FSF (−0.05 ± 0.12). Tukey’s post hoc test revealed significant differences between all pairs of materials for ΔE_00_ and ΔTP, whereas for ΔWID, no significant differences were found between GDA and EBF or between GUI and NSF (*p* > 0.05).

*Within-material changes*. Aging resulted in ΔE_00_ values exceeding the acceptability threshold (1.8) in OBF, CBF, EBF, and GUI, whereas the values of GDA and NSF were between the perceptibility and acceptability thresholds, and that of FSF remained below the perceptibility threshold. WID values decreased significantly after aging in all materials except FSF, in which a slight but significant increase was observed (*p* < 0.05). TP values decreased significantly in all materials except FSF, in which no significant change was found (*p* > 0.05) (repeated-measures ANOVA).

### 3.2. Effects of Staining and Bleaching on Optical Properties

*Between-material comparisons.* Significant differences among materials were found for all optical parameters at all treatment intervals (one-way ANOVA, *p* < 0.001; Table 2).

For ΔE_00_, Tukey’s post hoc test revealed significant differences between all pairs of materials in all three intervals. After staining, ΔE_00_ values ranged from 9.57 ± 0.09 (GDA) to 25.39 ± 0.08 (CBF), with CBF followed by NSF (15.74 ± 0.09), OBF (14.95 ± 0.09), EBF (14.76 ± 0.11), FSF (14.07 ± 0.10), and GUI (12.43 ± 0.06). The magnitude of color change produced by bleaching (ΔE_00_ Bleaching–Staining) ranged from 7.36 ± 0.04 (GDA) to 21.82 ± 0.09 (CBF), with CBF followed by EBF (13.79 ± 0.09), NSF (12.17 ± 0.07), FSF (11.85 ± 0.09), GUI (10.64 ± 0.08), and OBF (9.60 ± 0.07). The residual color difference relative to baseline (ΔE_00_ Bleaching–Baseline) ranged from 2.17 ± 0.07 (EBF) to 6.24 ± 0.09 (OBF), with OBF followed by NSF (4.36 ± 0.08), CBF (3.76 ± 0.08), GUI (2.78 ± 0.07), FSF (2.52 ± 0.07), and GDA (2.31 ± 0.09).

For ΔWID, no significant differences were found between GDA and GUI or between EBF and NSF in the Staining–Baseline comparison, or between GUI and EBF in the Bleaching–Baseline comparison (*p* > 0.05), whereas all other pairs differed significantly. The greatest loss of whiteness after staining was observed in CBF, followed by OBF, whereas GDA and GUI exhibited the smallest reductions.

For ΔTP, no significant differences were found between GDA and CBF or between EBF and NSF in the Bleaching–Staining comparison, or between GDA and NSF in the Bleaching–Baseline comparison (*p* > 0.05), whereas all other pairs differed significantly (Table 2).

*Within-material changes.* Staining decreased L* values (ΔL* from −12.83 to −27.95) and increased a* values in all materials, and increased b* values in all materials except GUI. Bleaching increased L* values and decreased a* and b* values in all materials. In all materials, the residual color difference relative to baseline was lower than the color difference after staining, indicating a reduction in discoloration, with the largest reduction observed in CBF (from 25.39 to 3.76). However, all materials remained darker than at baseline (ΔL* from −2.20 to −7.51), and ΔE_00_ Bleaching–Baseline values exceeded the acceptability threshold (1.8) in all materials (Table 2).

Staining significantly decreased WID and TP values in all materials, and subsequent bleaching significantly increased both parameters in all materials (repeated-measures ANOVA, *p* < 0.001). Compared with baseline, WID values after bleaching were significantly higher in GUI and EBF, whereas they remained significantly lower in the other materials, including both one-shade composites. Relative to baseline, the final TP values remained within the perceptibility threshold (±2 units) in CBF, GUI, and FSF. In contrast, translucency exceeded baseline values beyond the perceptibility threshold in OBF (+3.35) and EBF (+2.93), and a perceptible residual translucency loss was observed in GDA (−2.25) and NSF (−2.15).

## 4. Discussion

The methodological design of the present study was structured to ensure a standardized evaluation of optical changes in injectable resin composites. Disc-shaped specimens of uniform thickness were used to minimize geometric variability, allowing valid inter-material comparisons under controlled conditions [11,12,23]. Polishing was performed after a 24 h storage period to allow completion of post-polymerization reactions and to better reflect the optical behavior of mature composite surfaces [5]. Measurements were obtained using a calibrated dental spectrophotometer, and translucency was derived from the color difference between measurements over black and white backgrounds [24]. Coffee immersion for 12 days was used to simulate approximately one year of beverage consumption [23], and bleaching was applied after staining to reproduce a common clinical sequence, since in-office bleaching agents may affect resin composites in a material-dependent manner [23]. Injectable composites were selected because their optical outcomes may not be directly inferred from conventional paste composites, and one-shade systems may behave differently from group- and multi-shade materials owing to their shade-matching concept [4,12,30,31].

In the present study, the optical properties of injectable resin composites with different filler types and shade-matching strategies were evaluated following aging, coffee staining, and subsequent bleaching. The first, second, and third null hypotheses were rejected, as color, translucency, and whiteness changes differed significantly among the tested materials, indicating that the optical response to these procedures is material-dependent.

When interpreted in relation to established perceptibility and acceptability thresholds, aging and staining frequently resulted in ΔE_00_ values exceeding both the perceptibility (0.8) and acceptability (1.8) thresholds. Although bleaching substantially reduced the color differences, the residual color difference relative to baseline remained above the acceptability threshold in all materials. Similarly, ΔWID values exceeded the perceptibility threshold (0.72) and, in several materials, the acceptability threshold (2.60). ΔTP values exceeded the perceptibility threshold of 2 units in most materials after aging and staining. Thus, statistically significant changes after bleaching did not necessarily correspond to a return to baseline within the acceptability limits.

Artificial aging induced significant changes in the optical behavior of all tested materials. The multi-shade nanofilled FSF, the group-shade nanohybrid NSF, and the conventional microhybrid GDA exhibited the lowest color changes, remaining below the acceptability threshold, whereas the one-shade composites OBF and CBF showed the highest values. Aging promotes water sorption, resin matrix degradation, polymer plasticization, and alterations at the filler–matrix interface, which reduce light transmission and increase internal light scattering [8,9]. These results suggest that optical stability depends on several material-related factors, including resin matrix chemistry and the integrity of filler–matrix interactions. Shade-dependent color stability has also been reported for bulk-fill injectable resin composites [32].

Coffee staining produced pronounced color changes in all materials, with the one-shade nanohybrid CBF exhibiting the highest discoloration and both one-shade composites showing the greatest whiteness loss. Coffee induces discoloration through both surface adsorption and absorption of pigments into the resin matrix [5,6,7], and differences in resin matrix hydrophilicity, filler loading, and filler distribution affect water sorption and pigment penetration. Material-dependent and persistent discoloration of flowable and bulk-fill resin composites has similarly been reported [33,34,35]. It has been suggested that single-shade composites, which rely predominantly on structural color formation and controlled light scattering rather than conventional pigmentation, may be more sensitive to minor alterations in translucency and internal light transmission [4,36]. However, the tested commercial materials differ simultaneously in resin matrix composition, filler architecture, filler loading, and shade-matching strategy. Therefore, the present findings cannot isolate the independent contribution of shade-matching strategy, and these mechanisms should be regarded as possible explanations rather than demonstrated causes.

Resin matrix composition and filler type may also have contributed to the observed differences. Although Bis-GMA containing systems have a highly cross-linked structure, they may demonstrate increased susceptibility to water uptake, whereas Bis-GMA free systems display more variable optical behavior depending on their specific monomer formulations [6,7]. Filler architecture influences light scattering at the filler–matrix interface and refractive index compatibility. The reductions in translucency observed after aging and staining can be attributed to increased light scattering caused by water sorption, pigment accumulation, and degradation of the filler–matrix interface [8,9,10,12].

Bleaching produced substantial color changes in all materials, accompanied by increases in L* and decreases in a* and b* values, indicating that the color change was predominantly directed toward the baseline color, mainly through the recovery of lightness. The residual color differences relative to baseline were markedly lower than those after staining, most notably in CBF. However, the residual color differences exceeded the acceptability threshold (ΔE_00_ > 1.8) in all materials, and all materials remained darker than at baseline. This suggests that staining-related changes are not confined to superficial pigment adsorption but may involve matrix-related alterations that are not fully reversed by bleaching. This finding is consistent with previous studies demonstrating that bleaching agents primarily remove superficial extrinsic stains without consistently restoring baseline optical properties [10,12,13,14,15,30,31,37].

In contrast, clinically acceptable color outcomes after in-office bleaching have been reported for single-shade resin composites [38]. Several methodological differences may explain this discrepancy. First, single-shade composites from different manufacturers differ in resin matrix and filler formulations, and their responses to staining and bleaching cannot be assumed to be equivalent. Second, specimen thickness and background conditions strongly influence the measured color of highly translucent materials. Third, the staining protocol used in the present study produced severe discoloration (ΔE_00_ up to 25.39), so that even a substantial reduction after bleaching left residual differences above the acceptability threshold, whereas studies using milder staining or no staining start from considerably smaller color differences. Fourth, bleaching regimens differ in peroxide concentration, application time, and number of sessions. Finally, outcome definitions vary among studies. The present study evaluated the residual color difference of each specimen relative to its own baseline after staining and bleaching, whereas other studies have assessed color matching with the surrounding tooth structure or used different color difference formulas and thresholds. These differences limit direct comparisons between studies.

Translucency increased after bleaching in all materials, in a manner dependent on both resin matrix composition and filler architecture [12,14,15,16]. However, the final translucency relative to baseline differed among materials. In CBF, GUI, and FSF, the final TP values remained within the perceptibility threshold relative to baseline, whereas GDA and NSF retained a perceptible translucency loss. In OBF and EBF, translucency exceeded baseline values beyond the perceptibility threshold. This indicates that bleaching did not restore the original translucency of these materials but resulted in perceptibly higher translucency than at baseline. Changes in whiteness generally followed the trends observed for color differences. Staining reduced whiteness in all materials, whereas the changes after bleaching were material-dependent. Because perceived whiteness is more strongly influenced by changes in lightness and yellowness than by overall color difference, comparable ΔE_00_ values may correspond to markedly different ΔWID outcomes [5,14].

This study has certain limitations. As an in vitro investigation, the experimental conditions cannot fully replicate the complexity of the oral environment. Aging and staining/bleaching were performed on separate specimen subgroups. Therefore, the cumulative effects of aging, staining, and bleaching on the same specimens were not investigated, and the findings do not represent the combined effects of these procedures under a single sequential protocol. In addition, the tested commercial materials differ simultaneously in several characteristics, including resin matrix composition, filler architecture, filler loading, and shade-matching strategy, so the independent effect of each characteristic cannot be determined. Only one staining agent and a single in-office bleaching protocol were evaluated. Furthermore, although specimen thickness was verified prior to finishing and polishing, it was not re-measured after the procedure. Since the protocol was standardized and performed by a single operator, any minimal material loss would be expected to be comparable across groups [39,40,41,42,43,44,45,46]; however, slight variations in final specimen thickness cannot be completely excluded. Further studies using sequential protocols and materials differing in a single characteristic are required to clarify these effects.

## 5. Conclusions

Within the limitations of this in vitro study, the following conclusions can be drawn:

(1) The multi-shade nanofilled FSF, the group-shade nanohybrid NSF, and the conventional microhybrid GDA demonstrated the greatest color stability after artificial aging, whereas the one-shade composites OBF and CBF showed the highest color changes.
(2) After coffee staining, the one-shade nanohybrid CBF exhibited the highest color change, and both one-shade composites showed the greatest whiteness loss.
(3) Bleaching reduced staining-induced discoloration in all materials, most markedly in CBF; however, the residual color differences relative to baseline remained above the acceptability threshold in all tested materials.
(4) Bleaching increased translucency in all materials; in OBF and EBF, the final translucency exceeded baseline values beyond the perceptibility threshold, indicating that the original translucency was not restored.

Since the tested materials differ in several compositional characteristics, these differences should be attributed to the individual materials rather than to the shade-matching strategy alone.

## Figures and Tables

**Table 1:** Experimental groups: shade-matching strategy and shade, product name, group code, filler type, composition (filler; resin matrix), filler size, filler content (wt%/vol%), manufacturer, and lot number.

Shade-Matching Strategy/Shade	Material	Code	Filler Type	Composition (Filler; Resin Matrix)	Filler Size	Filler Content (wt%/vol%)	Manufacturer	Lot No.
One-shade	Omnichroma Bulk Fill Flow	OBF	Supra-nanospherical	SiO_2_–ZrO_2_ clusters; UDMA, TEGDMA	~260 nm	69/55	Tokuyama Dental, Tokyo, Japan	033M45
	Charisma Bulk Flow ONE	CBF	Nanohybrid	Ba–Al–F silicate glass, YbF_3_, SiO_2_; UDMA, EBADMA	0.02–5 μm	65/41	Kulzer, Hanau, Germany	N010035
Group-shade/Body A1	Estelite Bulk Fill Flow	EBF	Supra-nanospherical	SiO_2_–ZrO_2_; Bis-GMA, Bis-MPEPP, TEGDMA	200 nm	70/56	Tokuyama Dental, Tokyo, Japan	134E15
	Neo Spectra ST Flow	NSF	Nanohybrid	Spherical granulated barium–aluminum–borosilicate glass, ytterbium fluoride; Bis-EMA, DDDMA, urethane-modified Bis-GMA	0.1–3.0 μm	62.5/40	Dentsply, Konstanz, Germany	2409000694
Multi-shade/Body A1	G-ænial Universal Injectable	GUI	Nanofilled	Silica, barium glass; nanofill dimethacrylate monomer	150 nm	69/46	GC Europe, Leuven, Belgium	250220A
	Filtek Supreme Flowable Restorative	FSF	Nanofilled	Ytterbium trifluoride filler, non-agglomerated/non-aggregated surface-modified silica filler, surface-modified aggregated zirconia/silica cluster filler; Bis-GMA, TEGDMA, Procrylat resins	4–20 nm	65/46	3M ESPE Dental Products, St. Paul, MN, USA	11269495
Conventional/Body A1	Gradia Direct Anterior	GDA	Microhybrid	Silica, prepolymerized fillers; UDMA, methacrylate monomers	0.85 μm	73/64	GC Europe, Leuven, Belgium	210210C

Bis-EMA, ethoxylated bisphenol A dimethacrylate; Bis-GMA, bisphenol A-glycidyl methacrylate; Bis-MPEPP, 2,2-bis(4-methacryloxypolyethoxyphenyl)propane; DDDMA, 1,12-dodecanediol dimethacrylate; EBADMA, ethoxylated bisphenol A dimethacrylate; TEGDMA, triethylene glycol dimethacrylate; UDMA, urethane dimethacrylate; YbF_3_, ytterbium trifluoride; wt%, weight percent; vol%, volume percent.

**Table 2:** Changes in color (ΔE_00_), whiteness index (ΔWID), and translucency parameter (ΔTP) after staining and bleaching procedures (mean ± standard deviation, *n* = 10).

Group	ΔE_00_			ΔWID			ΔTP		
	Staining–Baseline	Bleaching–Staining	Bleaching–Baseline	Staining–Baseline	Bleaching–Staining	Bleaching–Baseline	Staining–Baseline	Bleaching–Staining	Bleaching–Baseline
OBF One-shade, supra-nanospherical	14.95 ± 0.09 ^e^	9.60 ± 0.07 ^b^	6.24 ± 0.09 ^g^	−39.02 ± 0.32 ^b^	29.13 ± 0.28 ^e^	−9.89 ± 0.30 ^a^	−10.96 ± 0.13 ^a^	14.31 ± 0.14 ^e^	3.35 ± 0.16 ^f^
CBF One-shade, nanohybrid	25.39 ± 0.08 ^g^	21.82 ± 0.09 ^g^	3.76 ± 0.08 ^e^	−52.87 ± 0.43 ^a^	45.11 ± 0.42 ^g^	−7.76 ± 0.34 ^b^	−5.65 ± 0.11 ^e^	6.48 ± 0.13 ^c^	0.83 ± 0.13 ^d^
EBF Group-shade, supra-nanospherical	14.76 ± 0.11 ^d^	13.79 ± 0.09 ^f^	2.17 ± 0.07 ^a^	−29.53 ± 0.29 ^c^	30.47 ± 0.29 ^f^	+0.94 ± 0.26 ^f^	−2.26 ± 0.13 ^g^	5.19 ± 0.11 ^b^	2.93 ± 0.12 ^e^
NSF Group-shade, nanohybrid	15.74 ± 0.09 ^f^	12.17 ± 0.07 ^e^	4.36 ± 0.08 ^f^	−29.33 ± 0.30 ^c^	26.75 ± 0.30 ^d^	−2.58 ± 0.25 ^d^	−7.18 ± 0.12 ^d^	5.03 ± 0.11 ^b^	−2.15 ± 0.12 ^a^
GUI Multi-shade, nanofilled	12.43 ± 0.06 ^b^	10.64 ± 0.08 ^c^	2.78 ± 0.07 ^d^	−13.17 ± 0.27 ^e^	13.80 ± 0.25 ^b^	+0.63 ± 0.20 ^f^	−7.98 ± 0.12 ^c^	8.31 ± 0.10 ^d^	0.33 ± 0.11 ^c^
FSF Multi-shade, nanofilled	14.07 ± 0.10 ^c^	11.85 ± 0.09 ^d^	2.52 ± 0.07 ^c^	−26.67 ± 0.33 ^d^	24.86 ± 0.36 ^c^	−1.82 ± 0.25 ^e^	−4.50 ± 0.10 ^f^	3.37 ± 0.10 ^a^	−1.12 ± 0.12 ^b^
GDA Conventional, microhybrid	9.57 ± 0.09 ^a^	7.36 ± 0.04 ^a^	2.31 ± 0.09 ^b^	−13.57 ± 0.32 ^e^	8.74 ± 0.22 ^a^	−4.83 ± 0.27 ^c^	−8.65 ± 0.09 ^b^	6.41 ± 0.10 ^c^	−2.25 ± 0.11 ^a^

Different superscript lowercase letters within the same column indicate statistically significant differences among materials (one-way ANOVA followed by Tukey’s HSD test, *p* < 0.05); letters were assigned in ascending order of mean values (a = lowest). OBF, Omnichroma Bulk Fill Flow; CBF, Charisma Bulk Flow ONE; EBF, Estelite Bulk Fill Flow; NSF, Neo Spectra ST Flow; GUI, G-ænial Universal Injectable; FSF, Filtek Supreme Flowable Restorative; GDA, Gradia Direct Anterior.

## Data Availability

The raw data supporting the conclusions of this article will be made available by the authors on request.

## Abbreviations

The following abbreviations are used in this manuscript:

ANOVA	Analysis of variance
Bis-GMA	Bisphenol A-glycidyl methacrylate
CBF	Charisma Bulk Flow ONE
CIELAB	Commission Internationale de l’Éclairage L*a*b* color space
CR	Contrast ratio
EBF	Estelite Bulk Fill Flow
FSF	Filtek Supreme Flowable Restorative
GDA	Gradia Direct Anterior
GUI	G-ænial Universal Injectable
HSD	Honestly significant difference
LED	Light-emitting diode
NSF	Neo Spectra ST Flow
OBF	Omnichroma Bulk Fill Flow
SD	Standard deviation
T0	Baseline measurement
T1	Measurement after aging or staining
T2	Measurement after bleaching
TP	Translucency parameter
WID	Whiteness index for dentistry
ΔE_00_	Color difference calculated using the CIEDE2000 formula
ΔTP	Change in translucency parameter
ΔWID	Whiteness index difference
